# Identification and validation of immune and prognosis-related genes in hepatocellular carcinoma: A review

**DOI:** 10.1097/MD.0000000000031814

**Published:** 2022-11-18

**Authors:** Yu-Yang Chen, Shi-Mao Zhang, Heng-Xia Zhao, Jing-Yue Zhang, Li-Rong Lian, De-Liang Liu, Shu-Fang Chu

**Affiliations:** a Shenzhen Bao’an Traditional Chinese Medicine Hospital Group, Shenzhen, Guangdong, People’s Republic of China; b Shenzhen Traditional Chinese Medicine Hospital, Shenzhen, People’s Republic of China; c The Fourth Clinical Medical College, Guangzhou University of Chinese Medicine, Shenzhen, People’s Republic of China.

**Keywords:** hepatocellular carcinoma, immune risk prognostic model, key genes, liver cancer

## Abstract

Bioinformatics methods were used to identify the key genes associated with the immune microenvironment of hepatocellular carcinoma (HCC) to construct an immune risk prognostic model (IRPM) and to study the correlation between IRPM’s risk groups and immune characteristics of patients with HCC.

**Methods:**

HCC transcriptome sequencing information was searched for immune-related genes (IRGs) that were regularly expressed in cancer tissues. The IRGs, which were strongly linked to overall survival were screened; the prognostic characteristics model was constructed using Cox regression analysis. IRPM’s independent prognostic value was explored; Kaplan–Meier survival and receiver-operating characteristic curves were used to determine the model prediction ability in the led-to queue.

**Results:**

Patients in the high-risk group (HRG) showed significantly poor outcomes. Gene Set Enrichment Analysis revealed factors involved in both the HRG and low risk group. Immune-related hub genes (IRHGs) and drug sensitivity expression levels revealed that all IRHGs were correlated with drug sensitivity for certain chemotherapy drugs.

**Conclusion:**

The study results may serve as a reference for improving prognosis, early screening, and immunotherapy in patients with HCC.

## 1. Introduction

Primary liver cancer commonly causes several deaths annually worldwide,^[1]^ and hepatocellular carcinoma (HCC) is a major form of liver cancer.^[2]^ Many factors contribute to HCC, including viral hepatitis, chemical carcinogens, polluted drinking water, tobacco and alcohol consumption, and genetic factors^[3]^; however, the specific pathogenesis is unclear. Patients with HCC have a poor prognosis because of the complex pathogenesis and high recurrence rate, and most patients have complications such as liver cirrhosis.^[4]^

In recent years, the rise of immunotargeted therapy has shed new light to cancer treatment, and immunotherapy is a rather active field of cancer research. With the development of medicine, we have a deeper understanding of cancer; however, many obstacles remain in the field of cancer immunotherapy. These include unpredictable reactions, treatment effects, the need for new biomarkers to assess the curative effect, and research on immune treatment-resistant mechanisms.^[5]^

Accurate prediction of immunoprognosis is important for treatment selection, but there is a lack of effective immunoprognosis model. Moreover, different individuals undergoing liver cancer treatment have different drug sensitivities to the same treatment and each regimen has a different sensitive or resistant population. Inappropriate treatment regimens can lead to initial drug resistance, prolong the optimal treatment time, and cause irreparable harm. Based on the above problems, the main objectives of this study were as follows: establish a nomogram prediction model to precisely predict the prognosis of immune genes in patients with HCC; verify immune-related models using external data and provide reliable data support for these models; correlations between the risk scores and immune infiltration were measured to understand the correlation between the risk scores and immune components; relationship between the model and drug sensitivity was analyzed to provide a theoretical basis for clinical treatment of liver cancer.

## 2. Patients and methods

### 2.1. Patient and data

We downloaded the RNA matrix of 424 samples from The Cancer Genome Atlas (TCGA) database, 50 of which were in the normal group and 374 were in the liver cancer group. Furthermore, we collected information on 370 patients with liver cancer in the clinics. Information and data collected from the ICGA website regarding 231 additional patients with liver cancer were used for external validation. We collected immune-related genes (IRGs) from ImmPort (http://www.immport.org/shared/home) and InnateDB (https://www.innatedb.com) databases. The IRGs are listed in Supplemental Digital Content (Table S1, http://links.lww.com/MD/H943).

### 2.2. Identification of IRHGs

We searched for differentially expressed genes (DEGs) in 50 normal tissues using the R package “Limma.” In total, 374 liver cancer tissues (False Discovery Rate < 0.05, |logFC| >1) were intermingled with the IRGs. Gene Ontology (GO) and Kyoto Encyclopedia of Genes and Genomes (KEGG) enrichment analyses of immune-related differential genes were performed using the R package “cluster Profiler.” The weighted gene co-expression network analysis (WGCNA) algorithm and LASSO regression were then used to define the IRHGs. First, Pearson correlation coefficients for all paired genes were calculated. Second, the power function was used to construct the adjacency matrix, and the power of β was set to 15 to guarantee a scale-free network. It was then transformed into a topological matrix with a topological overlap measure describing the degree of association between genes. Gene clustering was performed with a 1-topological overlap measure distance, and a dynamic pruning tree was constructed to identify the modules. Eventually, we drew a correlation heat map according to the correlation between the feature vectors and the tumor group and selected the first 2 modules of the MS score for the subsequent study.

Univariate Cox regression analysis was conducted to analyze the clinical data of patients, and LASSO Cox regression analysis was conducted to analyze the prognosis signature using the R package “glmnet.” The formula is:


$$\begin{array}{l} risk\;score=\;\Sigma \;coeffient\;\left(genei\right)\times expression\left(genei\right) \end{array}$$

The genes finally included in the model were defined as IRHGs.

### 2.3. Construction and validation of immune risk prognostic models (IRPMs)

Patients were divided into high-risk groups (HRGs) and low-risk groups (LRGs) based on risk scores using the R package “survminer.” Considering gene expression levels of the constructed IRPMs, the R package “Rtsne” and “GGplot2” package were used for principal component analysis (PCA) and T-SNE analyses to determine the distribution of different groups. The Kaplan–Meier survival (K–M) curve was plotted using the R package “survival” to assess the survival differences. The R package “time ROC” was used to plot the time-dependent receiver-operating characteristic (ROC) curve to assess the ability to predict the signature. K–M survival curves were then applied to determine differences in the survival of IRHGs in GEPIA2 (http://gepia2.cancer-pku.cn/). Moreover, univariate and multivariate Cox regression analyses combined with clinical information were used to determine the independent prognostic value of IRPMs.

### 2.4. External validation of IRPMs

Based on the same risk scoring formula, we validated the immune-related model in the International Cancer Genome Consortium (ICGC) cohort and used the K–M survival curve and ROC curve to explore the predictive ability of the model in the ICGC cohort. Finally, immune-related hub protein expression of genes was explored using the human protein mapping online database (http://www.proteinatlas.org).

### 2.5. Comprehensive analysis of characteristics and drug therapy of each immune risk subgroup

Based on the HRGs and LRGs with immune scoring risk, we used the Gene Set Variation Analysis package to conduct single-sample Gene Set Enrichment Analysis enrichment analysis to determine the differences in pathogenesis between the HRGs and LRGs. In the gene mutation analysis, we analyzed IRHGs based on the gene mutation information of 9 liver cancer datasets provided by cBioPortal. Using the R package “Maftools,” a waterfall diagram of the mutation load for both groups was created. To detect the association between the risk groups of immune-related prognostic models and the immune characteristics of patients with HCC, CIBERSORT was used to assess the differential expression of 22 types of immune cells in both groups. Based on the gene set on biological function constructed by Mariathasan et al,^[6]^ we compared the differences in tumor-associated processes between both groups and assessed the association of risk scores with immune invasion and tumor dryness by immune score, stroma score, and tumor dryness.

The CellMiner online website (https://discover.nci.nih.gov/cellminer) contains 60 different cells from 9 types of tumor cells in the department of NCI - 60 database. The correlation between IRG expression and drug sensitivity was studied using Pearson’s correlation analysis. A correlation analysis was performed for 263 drugs with FDA approval or clinical trials.

## 3. Results

### 3.1. IRHGs

**Figure 1.**
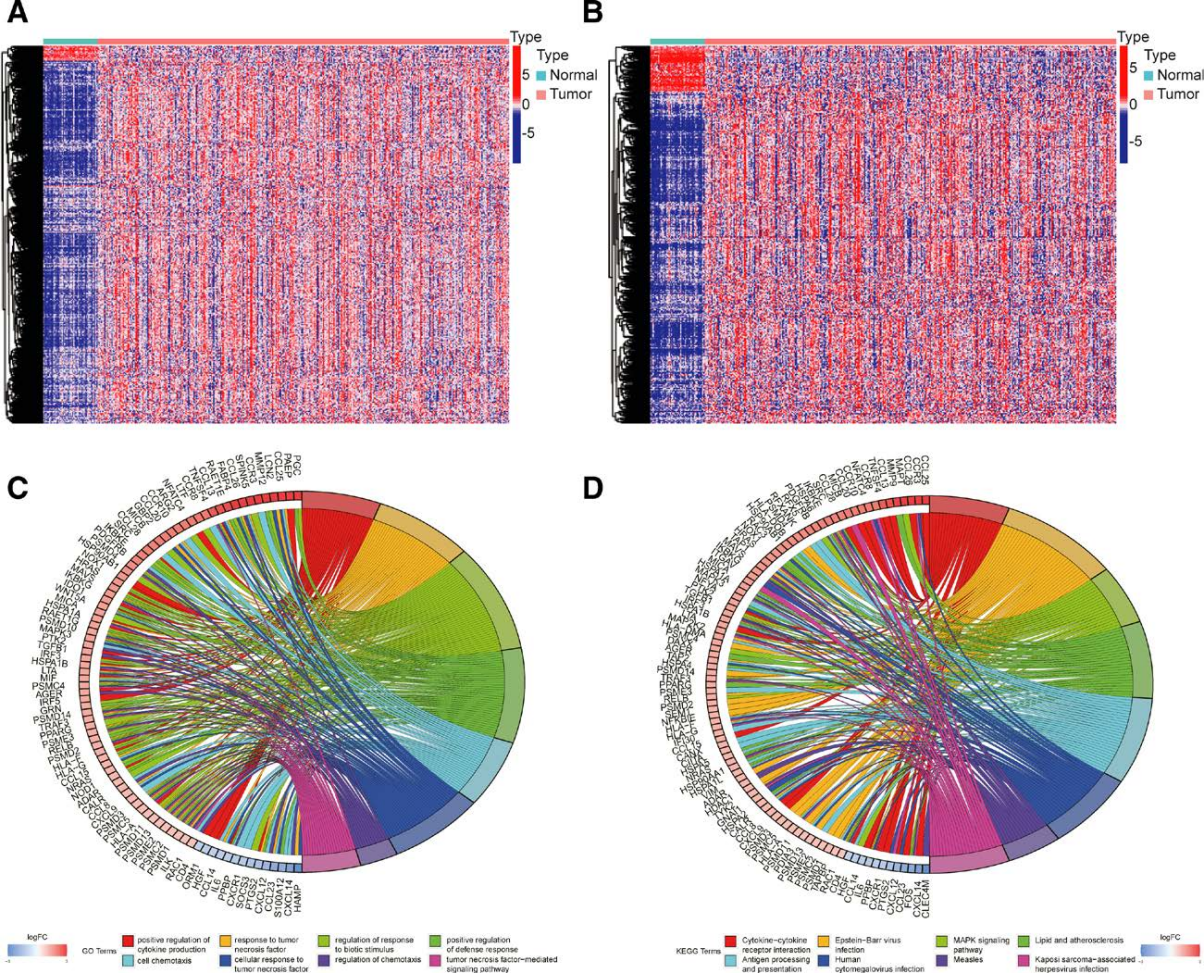
Gene expression differences between normal and liver cancer samples. (a, b) The 661 overlapping genes were all upregulated in tumor tissue. (c, d) Gene Ontology (GO) and Kyoto Encyclopedia of Genes and Genomes (KEGG) enrichment analyses indicated that 661 differentially expressed genes were significantly linked to immune-related functions and pathways.

**Figure 2.**
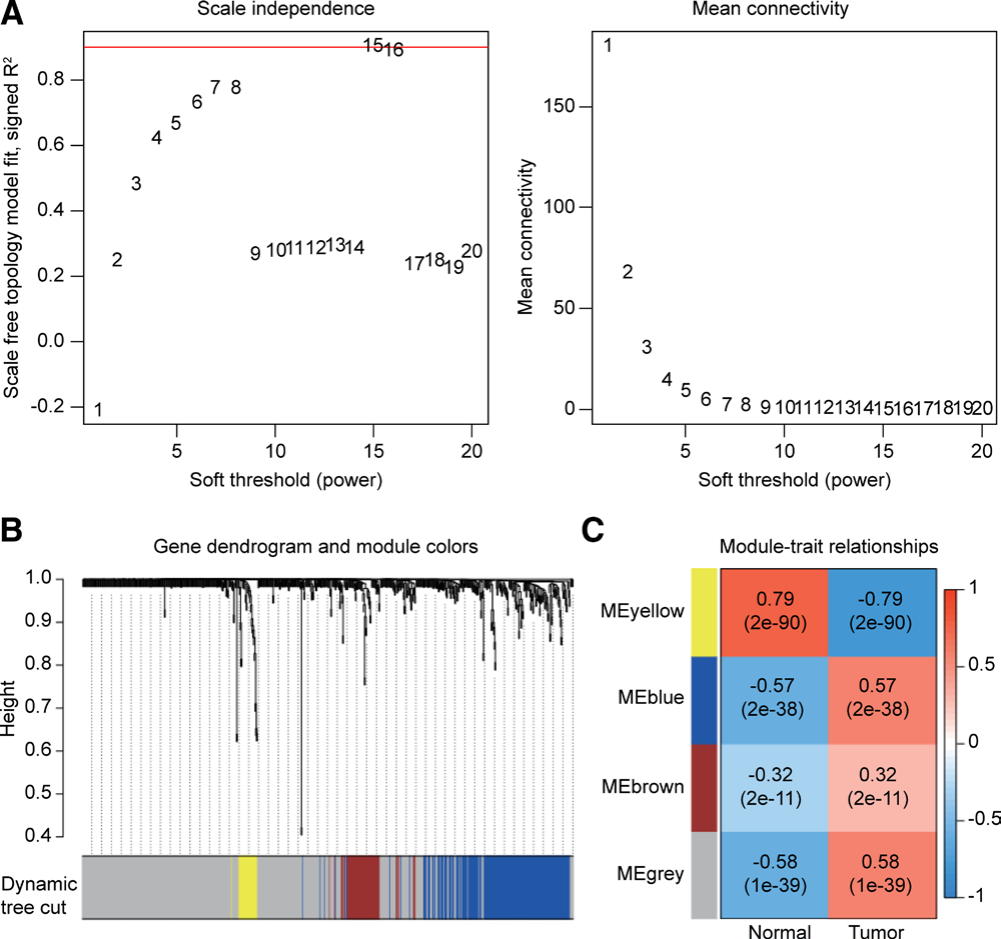
Identification of immune-related hub genes (IRHGs). (a) WGCAN algorithm and Lasso regression were used to define IRHGs. (b, c) Four modules were ultimately defined using the dynamic pruning tree, and the blue and gray modules were found to be significantly associated with the tumor.

By comparing gene expression differences between 50 normal tissue samples and 374 liver cancer samples, 7636 DEGs were ultimately screened. In total, 661 immune-related DEGs were defined through the intersection with the IRGs (Fig. 1a and b). GO and KEGG enrichment analyses indicated that 661 DEGs were significantly linked to immune-related functions and pathways (Fig. 1c and d). According to the results of WGCNA, when the soft threshold value β = 15, the correlation coefficient was > 0.9 (Fig. 2a). Four modules were ultimately defined using the dynamic pruning tree, and the blue and gray modules were found to be significantly associated with the tumor (Fig. 2b and c). The protein interaction networks of genes in the 2 modules were constructed using the STRING database, and the biological functions of the 2 modules were explored through GO and KEGG enrichment analyses (Supplemental Digital Content [Fig. S1, http://links.lww.com/MD/H945]).

**Figure 3.**
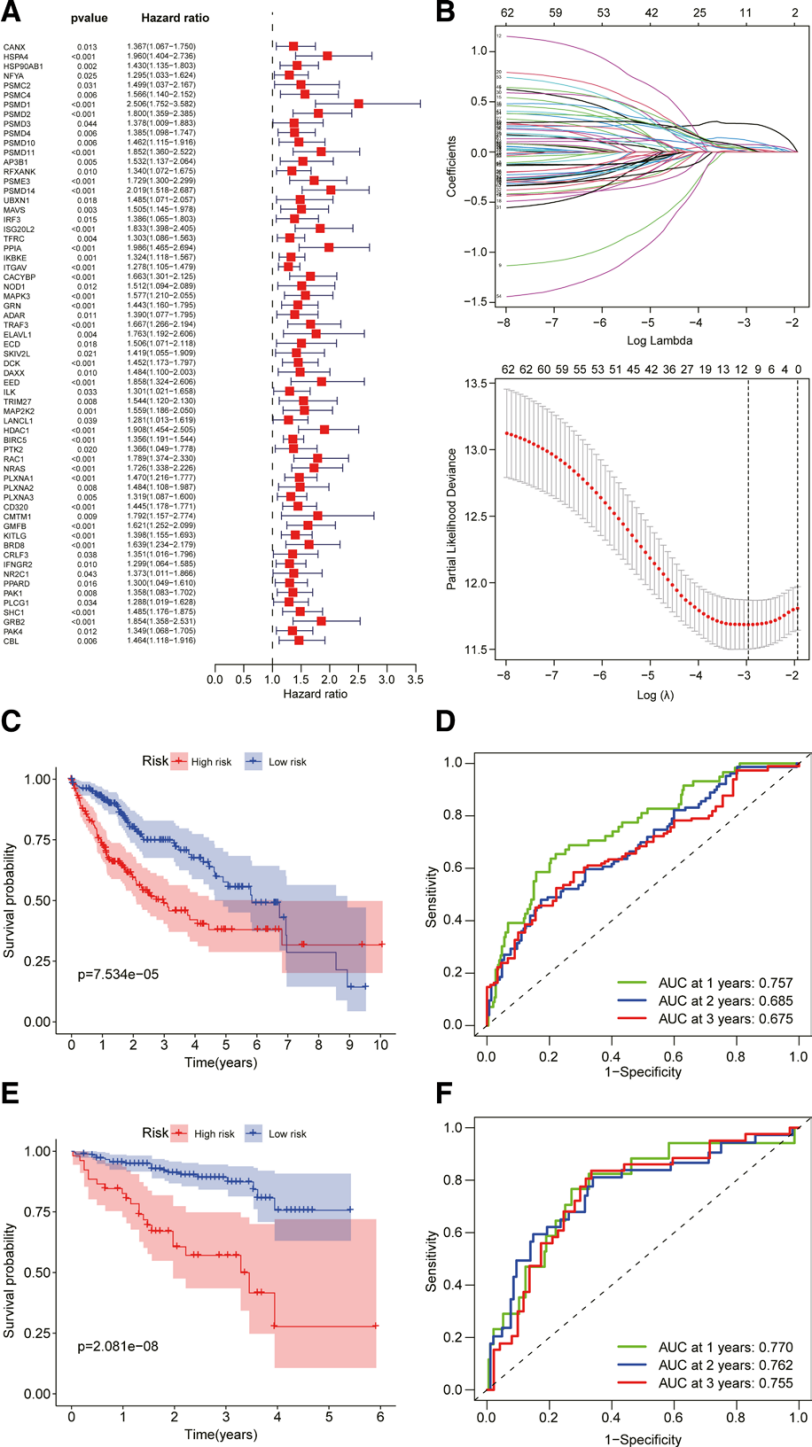
Acquiring immune-related hub genes (IRHGs) and constructing immune risk prognostic model (IRPM). (a) Univariate Cox regression analysis of genes in the 2 modules. (b) Lasso regression analysis of prognostic model. (c, e) Kaplan–Meier survival (K–M) curves for the overall survival (OS) of patients in the high-risk (HRGs) and low-risk groups (LRGs). (d, f) Area under the receiver-operating characteristic (ROC) curve (AUC) of time-dependent ROC curves in the international Cancer Genome Consortium (ICGC) cohort.

To acquire IRHGs for construction of the IRPM, survival analysis was performed on genes of the 2 modules, and the univariate Cox regression analysis showed that 63 genes were linked to overall survival (OS) (Fig. 3a). Subsequently, the prognostic model was constructed through LASSO regression analysis, and the markers of 10 genes were determined based on the optimum values of λ (Fig. 3b). The risk scores were calculated as follows: risk score = PSMD1 expression * 0.279820589858987 + PSMD14 expression * 0.0467515934356898 + ISG20L2 * 0.0357277361787815 + PPIA expression * 0.113470291570958 + HDAC1 * 0.0741004186265023 + BIRC5 expression * 0.0730458510345646 + RAC1 * 0.0216000708897467 + NRAS * 0.0255956540968313 + CD320 * 0.0352978307665251 + GMFB * 0.0227309038559405. Patients were split into HRGs and LRGs based on the median risk score (Supplemental Digital Content [Fig. S2a, http://links.lww.com/MD/H946]). Both the scatter plots (Supplemental Digital Content [Fig. S2b, http://links.lww.com/MD/H946]) and survival curves indicated a significantly poorer prognosis in patients with HRG (Fig. 3c). PCA and t-SNE analyses showed that gene expression levels of the high-risk and low-risk patients were distributed in different directions (Supplemental Digital Content [Fig. S2b, http://links.lww.com/MD/H946]). The area under the ROC curve (AUC) at 1–3 years was 0.757, 0.685, and 0.675, respectively (Fig. 3d), which demonstrated the good predictive ability of the model. To examine the stability of the 10 IRHGs prognostic models, an identical risk score formula was used to determine the risk scores of 231 patients in the ICGC cohort, and patients in the ICGC cohort were divided into HRGs and LRGs using the same median risk score as in the TCGA model (Supplemental Digital Content [Fig. S2c, http://links.lww.com/MD/H946]). The PCA and t-SNE analysis results confirmed the differential distribution of gene expression of HRGs and LRGs, which was similar to the results obtained with the TCGA cohort (Supplemental Digital Content [Fig. S2d, http://links.lww.com/MD/H946]). Patients with HRG also had a poorer prognosis than patients with LRG (Fig. 3e). The AUC values of the 10-gene signature at 1 to 3 years were 0.77, 0.762, and 0.755, respectively (Fig. 3f).

### 3.2. Construction and clinical characteristics of a novel prognostic nomogram

**Figure 4.**
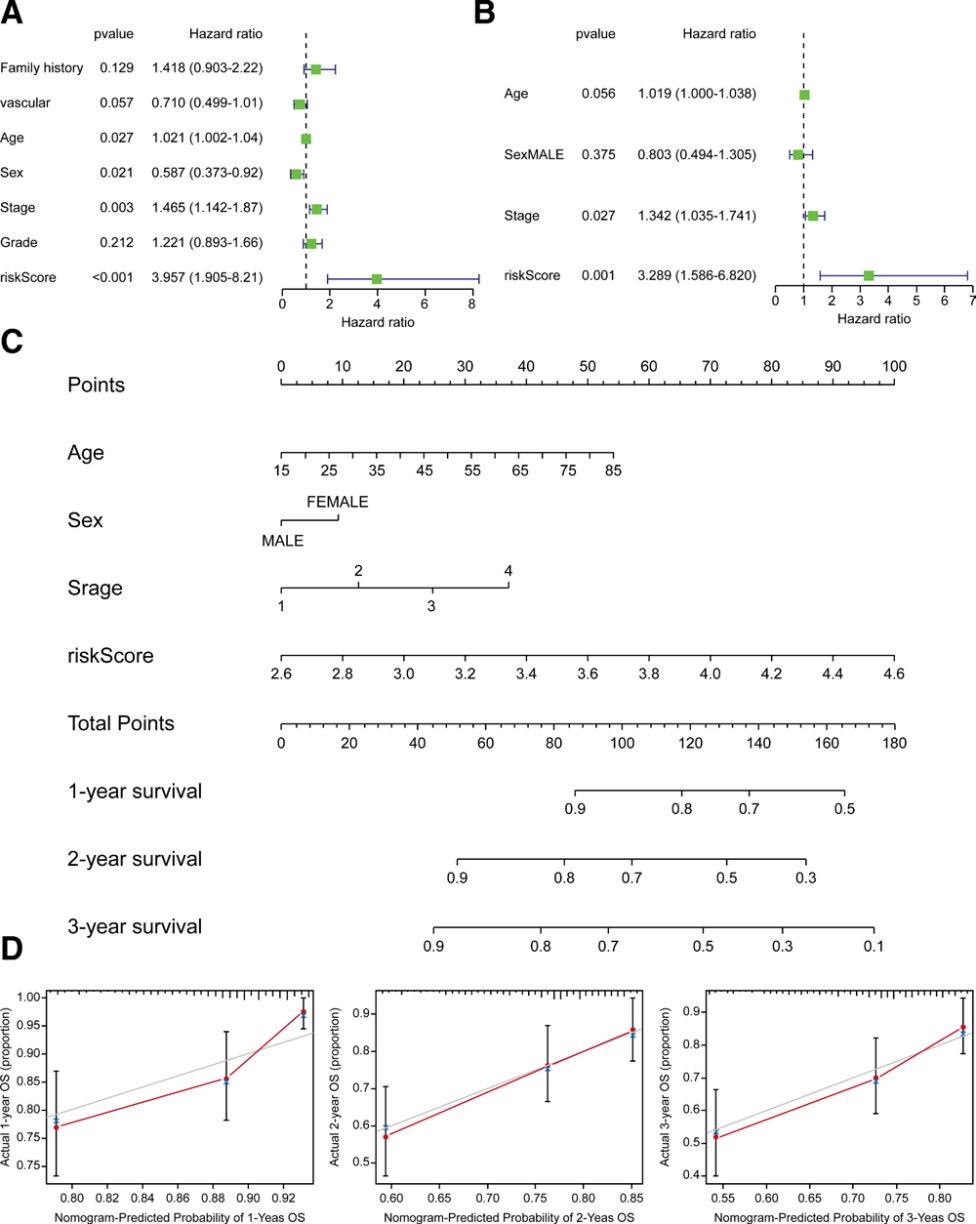
Correlations between the risk score and clinical characteristics of patients with hepatocellular carcinoma (HCC). (a) Univariate regression analysis was utilized with clinical information to assess whether the risk score was an independent prognostic factor. (b) Multivariate regression analysis was utilized with clinical information to assess whether the risk score was an independent prognostic factor. (c) Novel prognostic nomogram was constructed based on factors with *P* < .05 in univariate Cox regression. (d) Predictive ability of prognostic model.

By analyzing correlations between the risk score and clinical characteristics of patients with HCC, we found that patients with stage 2 or 3 tumors and grade 3 or 4 tumors had significantly higher risk scores. In comparison, patients with stage 1 tumors had significantly lower scores (Supplemental Digital Content [Fig. S3, http://links.lww.com/MD/H947]). Univariate (Fig. 4a) and multivariate regressions (Fig. 4b) were utilized with clinical information to assess whether the risk score was an independent prognostic factor. It was found that the risk score was closely related to OS even when multiple clinical factors (Supplemental Digital Content [Fig. S4, http://links.lww.com/MD/H948]), such as family history, age, and clinical stage, were considered. Ultimately, a novel prognostic nomogram was constructed based on factors with *P* < .05 in univariate Cox regression (Fig. 4c), and calibration curves at 1 to 3 years also demonstrated the good predictive ability of our prognostic model (Fig. 4d).

### 3.3. Validation of the 10 IRHGs using external data

**Figure 5.**
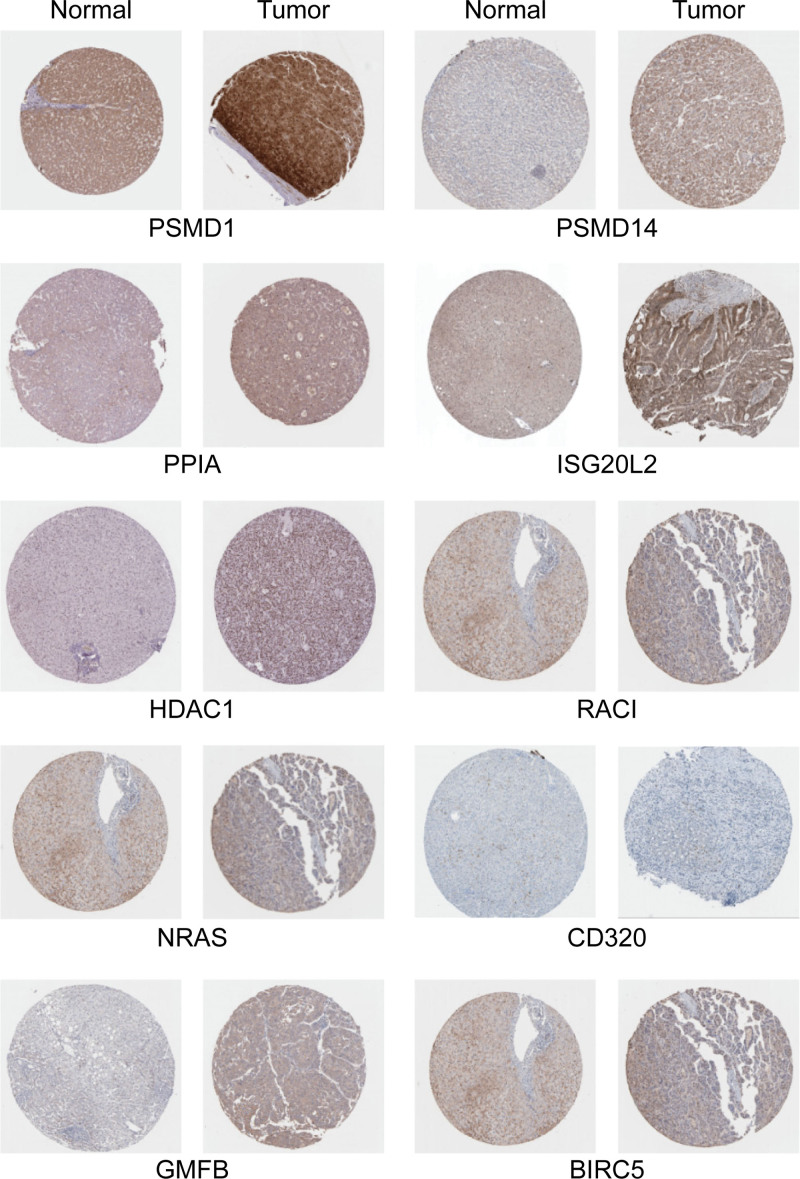
The protein level of 10 immune-related hub genes (IRHGs) in normal liver tissues and liver cancer samples from the Human Protein Atlas.

To validate the differential expression of the 10 IRHGs, we utilized data from the Human Protein Atlas to explore protein levels of IRHGs in normal liver tissues and liver cancer samples (Fig. 5). The prognostic value of these IRHGs was also explored using GEPIA2. The survival analysis results indicated that overexpression of the 10 IRHGs was closely associated with poor prognosis. Lastly, genomic changes in the IRHGs were investigated using cBioPortal. ISG20L2 (7%) exhibited the highest mutation rate, followed by BIRC5 (2.7%), PSMD1 (1.1%), PSMD14 (1.1%), HDAC1 (0.8%), NRAS (0.8%), PPIA (0.6%), RAC1 (0.6%), GMFB (0.4%), and CD320 (0.3%) (Supplemental Digital Content [Fig. S5, http://links.lww.com/MD/H949]).

### 3.4. Relationships of risk group and tumor microenvironment with biological function

**Figure 6.**
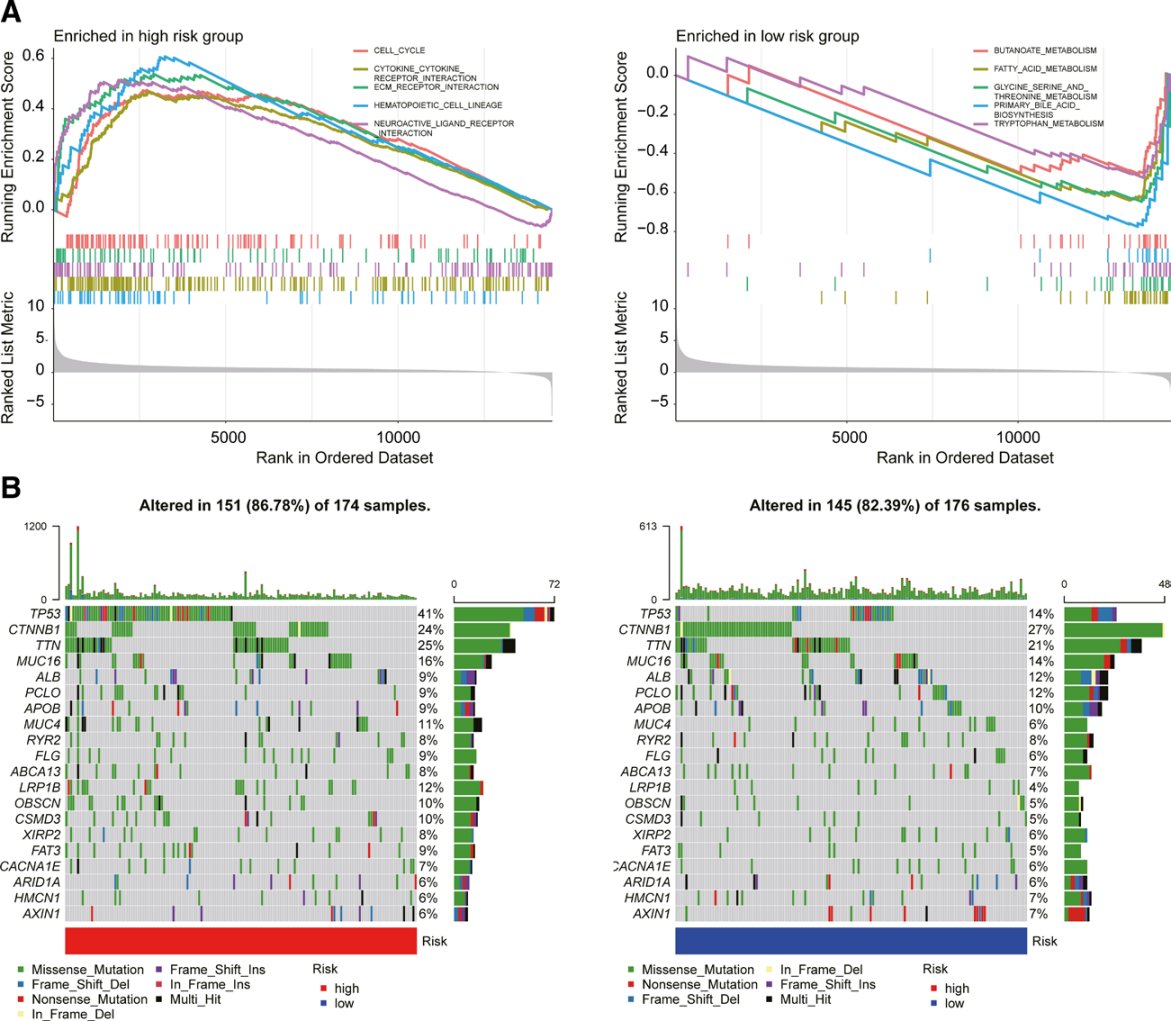
Comparison of the single-sample Gene Set Enrichment Analysis (GSEA) scores between different risks groups in the The Cancer Genome Atlas (TCGA) cohort. (a) GSEA enrichment analysis was conducted on both high-risk and low-risk groups (HRG and LRG, respectively). (b) Mutational burden analysis showed that TP53 mutations were predominant in the HRG while CTNNB1 mutations were predominant in the LRG.

Gene Set Enrichment Analysis enrichment analysis was conducted for both the HRGs and LRGs. The results indicated that “cell cycle, cytokine receptor interaction, hematopoietic cell lineage, and neuroactive ligand-receptor interaction” were mainly enriched in the HRG and “butanoate metabolism, fatty-acid-metabolism, glycine, serine, and threonine metabolism, primary bile acid biosynthesis, and tryptophan metabolism” were mainly enriched in the LRG (Fig. 6a). Mutational burden analysis showed that TP53 mutations were predominant in the HRG, whereas CTNNB1 mutations were predominant in the LRG (Fig. 6b).

**Figure 7.**
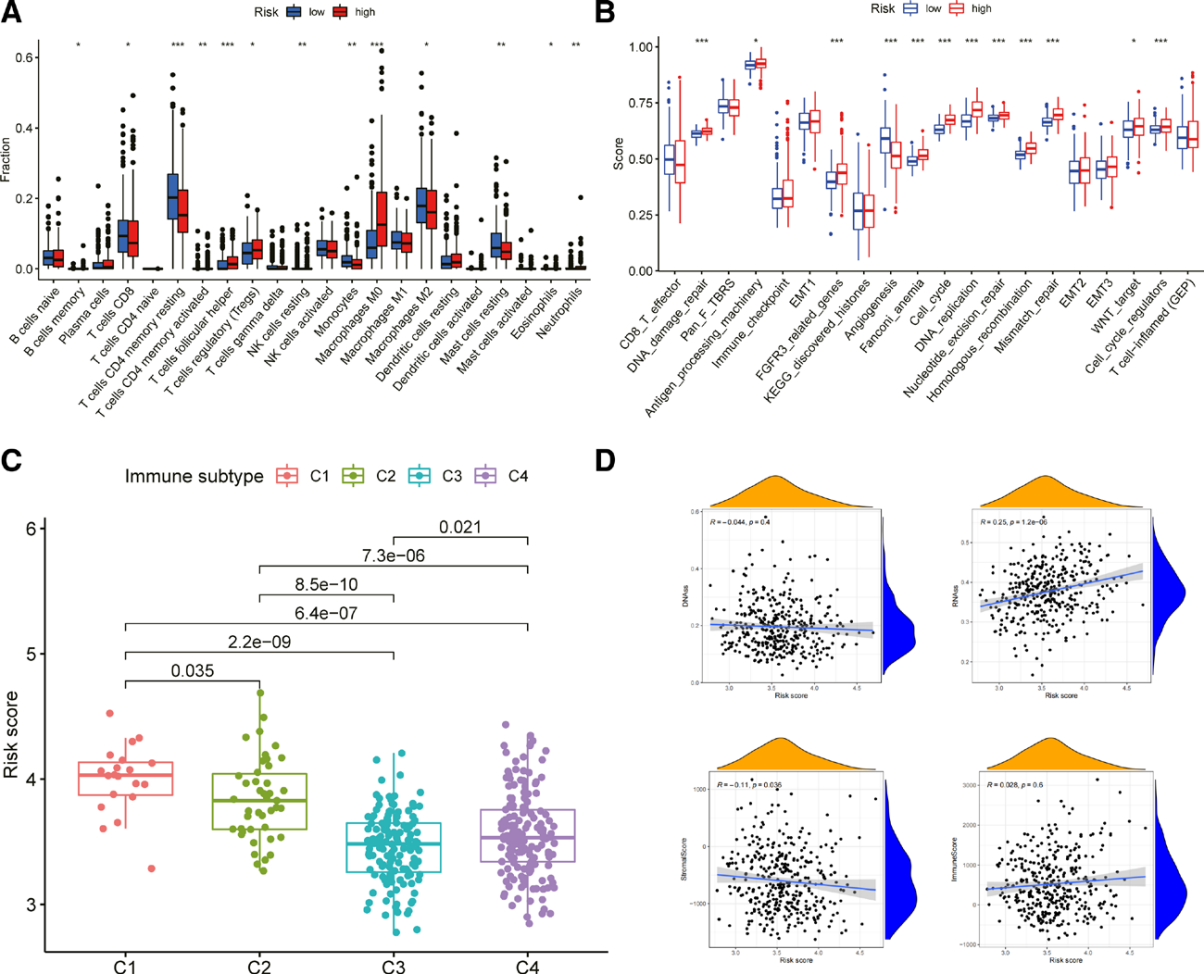
Relationships of risk group and tumor microenvironment with biological function. (a) Comparison of the CIBERSORT scores between risk type and immune characteristics. (b) Relationship between the risk score and immune components. (c) Relationships between data on immune infiltration in hepatocellular carcinoma (HCC) from the The Cancer Genome Atlas database-hepatocellular carcinoma (TCGA-HCC) cohort and the risk score. (d) Comparison of the RNA stemness scores (RNAss), DNA methylation mode-based DNA stemness scores (DNAss), stromal scores, and immune scores between high-risk and low-risk groups (HRG and LRG, respectively).

Next, to explore the relationships between risk type and immune characteristics, we determined the scores of 22 types of immune cells in patients with liver cancer using CIBERSORT. We found a different distribution of immune cells in HRGs and LRGs. For example, the expression of CD8 T cells and CD4 memory resting T cells in the LRG was significantly higher than that in the HRG, whereas the expression of regulatory T cells and M0 macrophages was higher in the HRG (Fig. 7a). Subsequently, we tested the correlations between the risk score and immune infiltration to elucidate the relationship between the risk score and immune components (Fig. 7b). Six subtypes of immune cell infiltration were identified in human tumors^[7]^: C1 (wound healing), C2 (INF-γ dominant), C3 (inflammation), C4 (lymphocyte exhaustion), C5 (immune silencing), and C6 (TGF-β dominant). When data on immune infiltration in HCC from the TCGA-HCC cohort were analyzed to determine relationships with the risk score, we found that high-risk scores were associated with C1, and low-risk scores were associated with C3 (Fig. 7c). The mRNA expression-based RNA stemness scores, DNA methylation mode-based DNA stemness scores,^[8]^ stromal scores, and immune scores were also analyzed to assess the differences in immune infiltration between HRGs and LRGs. Spearman’s correlation analysis revealed that the risk score had a strong positive correlation with the mRNA stemness score and a strong negative correlation with the stromal score (Fig. 7d).

**Figure 8.**
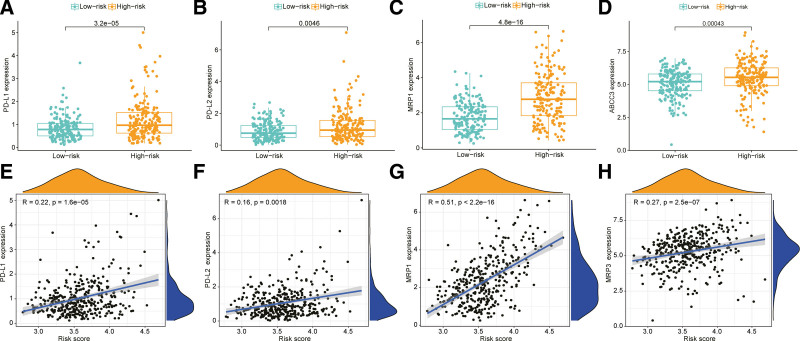
Relationships of risk group and tumor microenvironment with biological function. (a, b, e, f) Correlations between PD-L1 and PD-L2 expression levels and risk scores; (c, d, g, h) Correlations between MRP1 and ABCC3 expression levels and risk scores.

Recent studies reported PD-L1 overexpression and utilization of PD-L1/PD-1 signaling to evade T-cell immunity in many types of cancers. Therefore, expression levels of immune checkpoints, including PD-L1 and PD-L2, serve as key indicators for individualized immunotherapy.^[3]^ Our results indicated the presence of significant PD-L1 and PD-L2 overexpression in the HRG and significant positive correlations between PD-L1 and PD-L2 expression levels and risk scores (Fig. 8a, b, e, and f). Significant overexpression of the common drug resistance genes, MRP1 and MRP3 and significant positive correlations of their expression levels with the risk score were also observed in the HRG (Fig. 8c, d, g, and h).

### 3.5. Prediction of immunotherapy

**Figure 9.**
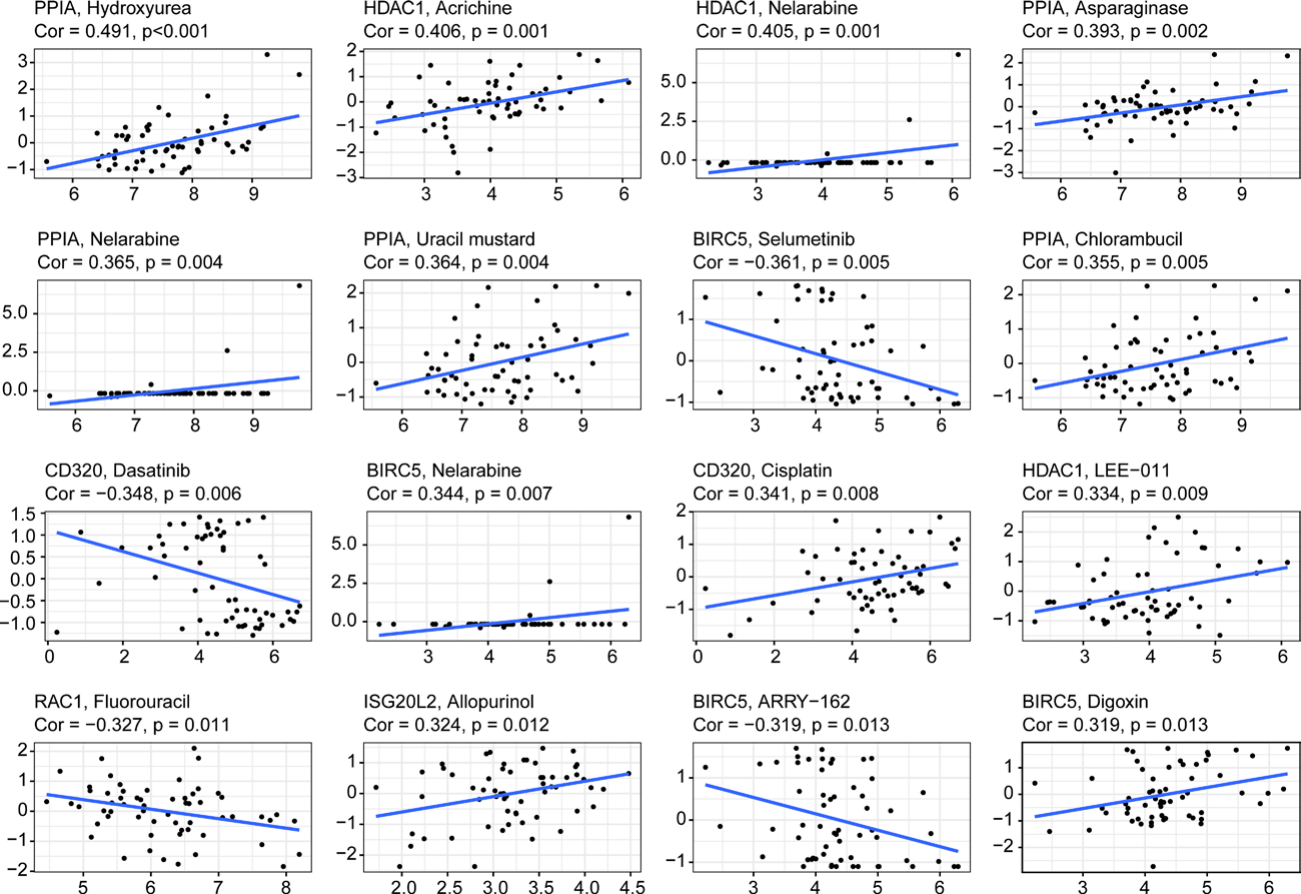
Relationship between expression levels of 10 IRHGs and drug sensitivity.

We analyzed the relationship between expression levels of 10 IRHGs and drug sensitivity. The results indicated that all IRHGs were correlated with sensitivity to certain chemotherapy drugs (*P* < .01) (Supplemental Digital Content [Table S2, http://links.lww.com/MD/H944]). From the correlation plots of the top 16 chemotherapy drugs (Fig. 9), it is observed that an increase in PPIA, HDAC1, and ISG20L2 expression was associated with increased drug sensitivity to hydroxyurea, acrichine, nelarabine, and allopurinol, whereas the expression of CD320, RAC1, and BIRC5 may be related to cancer resistance to dasatinib, fluorouracil, and ARRY-162.

## 4. Discussion

With advancement in targeted immunotherapy, the development of inhibitors targeting immune checkpoints has brought new hope to patients with cancer. However, there is still a lack of biomarkers that can be utilized in diagnosing liver cancer, necessitating the development of novel therapeutic targets to aid in clinical diagnosis. Moreover, we systematically analyzed the expression of 4909 IRGs in HCC tissues and their relationship with OS. Modules closely connected with tumor onset were identified using the WGCNA algorithm. Ultimately, 10 immune-related prognostic genes were determined and subsequently validated in an ICGC cohort through univariate Cox regression and LASSO regression. Patients were divided into HRGs and LRGs based on the median risk score. We found that HRG had a significant relationship with higher tumor grade, advanced TNM stage, and shorter OS. Independent prognostic analysis indicated that the risk score was an independent predictor of OS.

The IRPM developed in this study was established using 10 genes, namely PSMD1, PSMD14, ISG20L2, PPIA, HDAC1, BIRC5, RAC1, NRAS, CD320, and GMFB. These genes are significantly overexpressed in liver cancer and are closely related to poor prognosis in patients. Research has indicated that PSMD1 overexpression in gastric cancer leads to a poor prognosis.^[9]^ PSMD1 is also a potential and novel therapeutic target in anaplastic thyroid carcinoma and breast carcinoma tissues.^[10,11]^ Researchers have used the deubiquitinating enzyme, PSMD14 as a novel marker in liver cancer, as it can hasten the growth and metastasis of cancer cells through stabilization of GRB2.^[12]^ Many bioinformatics studies reported that ISG20L2 is closely associated with immune infiltration and survival in patients with liver cancer,^[4,13]^ making it a potential novel therapeutic target that warrants further validation using external data. In lung cancer and endometrial cancer, PPIA is significantly overexpressed and it causes poor prognosis in patients.^[2,14]^ Recent research has shown that HDAC1, a member of the class I histone deacetylases, plays an essential role in cellular senescence, liver aging, myelination, and adult neurogenesis,^[15]^ and its inactivation may induce liver cancer cell death.^[16]^ Cao et al^[1]^ found that OCT4 upregulates BIRC5 and CCND1 expression to promote HCC cell proliferation. RAC1 has been explored as a prognostic marker in HCC, and the Tiam1-Rac1 pathway significantly influences tumor progression in HCC.^[17]^ The RAS family, which includes NRAS, consists of oncogenes that are most frequently mutated in cancers. Research has shown that NRAS mutations in liver cancer are closely associated with tumor drug resistance.^[18]^ Quadros et al^[19]^ observed the overexpression of CD320 in multiple types of cancer and proposed that targeting the absorption of VB12 through the CD320 receptor and antibody-toxic conjugate may serve as a feasible treatment strategy for certain cancers with CD320 receptor overexpression. This is consistent with our research findings and indicates that CD320-targeted immunotherapy may become a novel targeted immunotherapy approach for liver cancer. There is insufficient research on GMFB in tumors, and the pathogenic mechanisms of this gene in liver cancer remain unclear.

In the HRG, exhaustion was observed in multiple types of T cells, which indicated disrupted immunomodulatory function in high-risk patients.

Our results showed that the risk score was strongly correlated with PD-L1 and PD-L2 expression levels. Consequently, the prognostic model could predict expression levels of immune checkpoints. Furthermore, it may provide guidance for the formulation of immunotherapy strategies. A high-risk score had a significant relationship with HCC pathways including DNA replication and cell cycle. This may be related to frequent DNA strand breakage caused by excessive tumor cell proliferation. Therefore, drugs targeted towards DNA damage repair may be significantly beneficial in patients with higher risk scores.

Using NCI-60 cell line data, we found that an increase in the expression of certain prognostic genes was associated with increased resistance to many FDA-approved chemotherapy drugs, including selumetinib, dasatinib, and fluorouracil. This may be due to some relationships between prognostic genes and sensitivity to certain drugs. Previous studies have shown that the multidrug resistance-associated protein family is closely related to increased drug resistance in patients.^[20]^ Our results showed that higher risk scores were strongly correlated with MRP1 and MRP3 expression, indicating that targeting tumor drug resistance genes may serve as a fundamental approach for patients with cancer. In summary, immune-related prognostic genes may play essential roles in improving prognosis.

## Author contributions

All authors contributed to the study conception and design. Material preparation, data collection, and analysis were performed by Yu-yang Chen and Shi-mao Zhang. The first draft of the manuscript was written by Yu-yang Chen and Shi-mao Zhang, and all authors commented on previous versions of the manuscript. All authors read and approved the final manuscript.

**Conceptualization:** Yu-Yang Chen, Shi-Mao Zhang.

**Data curation:** Yu-Yang Chen, Shi-Mao Zhang, Jing-Yue Zhang, Li-Rong Lian.

**Formal analysis:** Yu-Yang Chen, Shi-Mao Zhang.

**Funding acquisition:** Yu-Yang Chen, Shi-Mao Zhang, Jing-Yue Zhang, Li-Rong Lian.

**Investigation:** Yu-Yang Chen, Shi-Mao Zhang.

**Methodology:** Yu-Yang Chen, Shi-Mao Zhang.

**Project administration:** Heng-Xia Zhao, De-Liang Liu, Shu-Fang Chu.

**Resources:** Yu-Yang Chen, Shi-Mao Zhang.

**Software:** Yu-Yang Chen, Shi-Mao Zhang, Jing-Yue Zhang, Li-Rong Lian.

**Supervision:** Yu-Yang Chen, Shi-Mao Zhang.

**Validation:** Yu-Yang Chen, Shi-Mao Zhang.

**Visualization:** Yu-Yang Chen, Shi-Mao Zhang.

**Writing – original draft:** Yu-Yang Chen, Shi-Mao Zhang.

**Writing – review & editing:** Yu-Yang Chen, Shi-Mao Zhang.

## Supplementary Material
