# Supplementary material for: Identification and validation of immune and prognosis-related genes in hepatocellular carcinoma: A review
Source: Medicine (Baltimore). 2022 Nov 18;101(46):e31814. doi: 10.1097/MD.0000000000031814 (PMC9678506; doi:10.1097/MD.0000000000031814)

Figure S1. Biological functions of blue and grey modules were explored through Gene Ontology (GO) and Kyoto Encyclopedia of Genes and Genomes (KEGG) enrichment analyses

(a, b) The protein-protein interaction (PPI) network downloaded from the STRING database indicated the interactions among the blue and grey modules

(**c/d/e/f)** The most significant or shared GO enrichment and KEGG pathways in the blue and grey modules are displayed

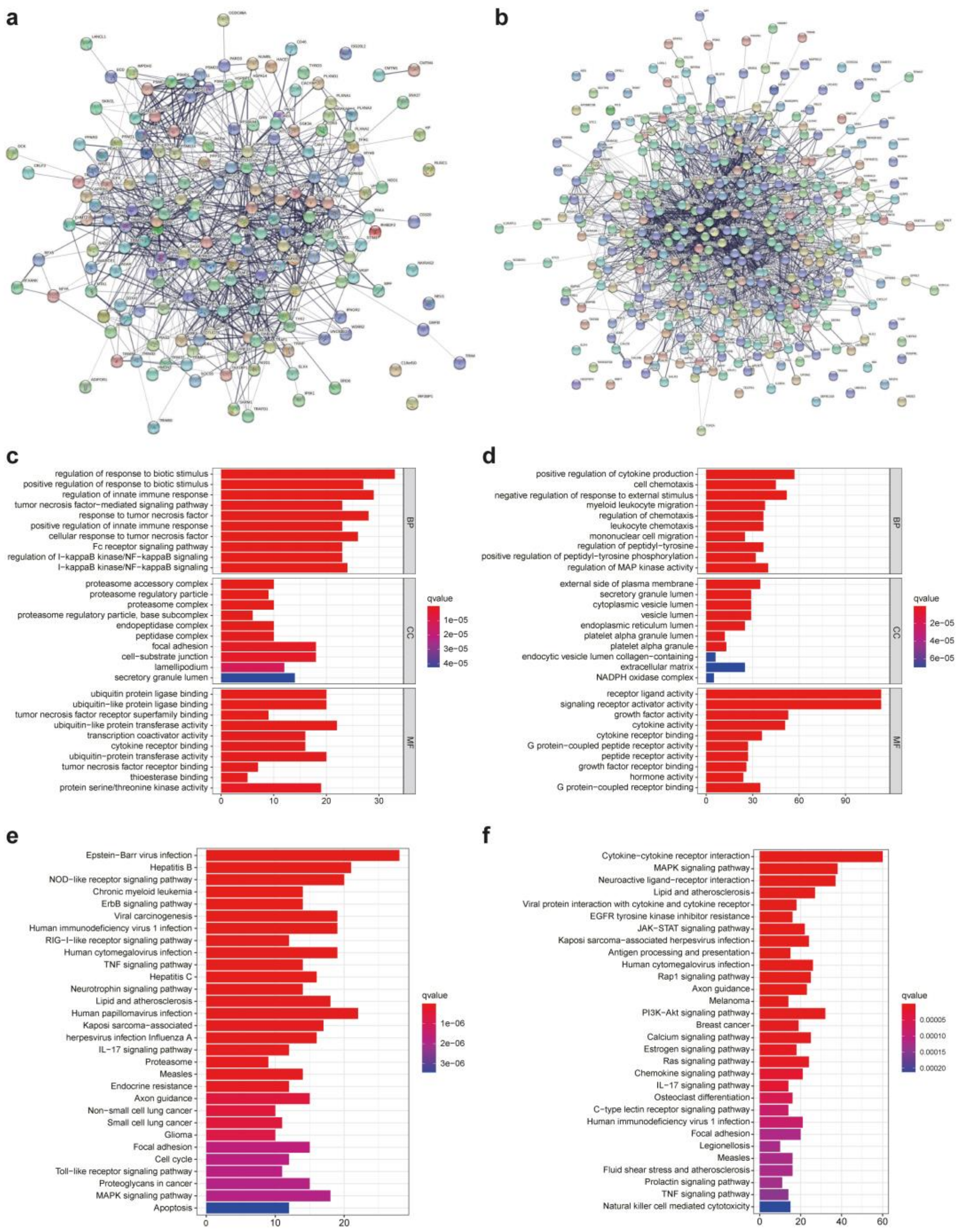

Supplement: Supplementary file 3 [file medi-101-e31814-s003.pdf]
