# Supplementary material for: Identification and validation of immune and prognosis-related genes in hepatocellular carcinoma: A review
Source: Medicine (Baltimore). 2022 Nov 18;101(46):e31814. doi: 10.1097/MD.0000000000031814 (PMC9678506; doi:10.1097/MD.0000000000031814)

Figure S2. Validation of the 10-gene signature

(**a**) The distribution and median value of the risk scores in The Cancer Genome Atlas (TCGA) cohort

(**b**) Principal component analysis (PCA) plot and t-SNE analysis of the TCGA cohort.

(**c**) The distribution and median value of the risk scores in the International Cancer Genome Consortium (ICGC) cohort. Dihedral principal component analysis (D PCA) plot and t-SNE analysis of the ICGC cohort

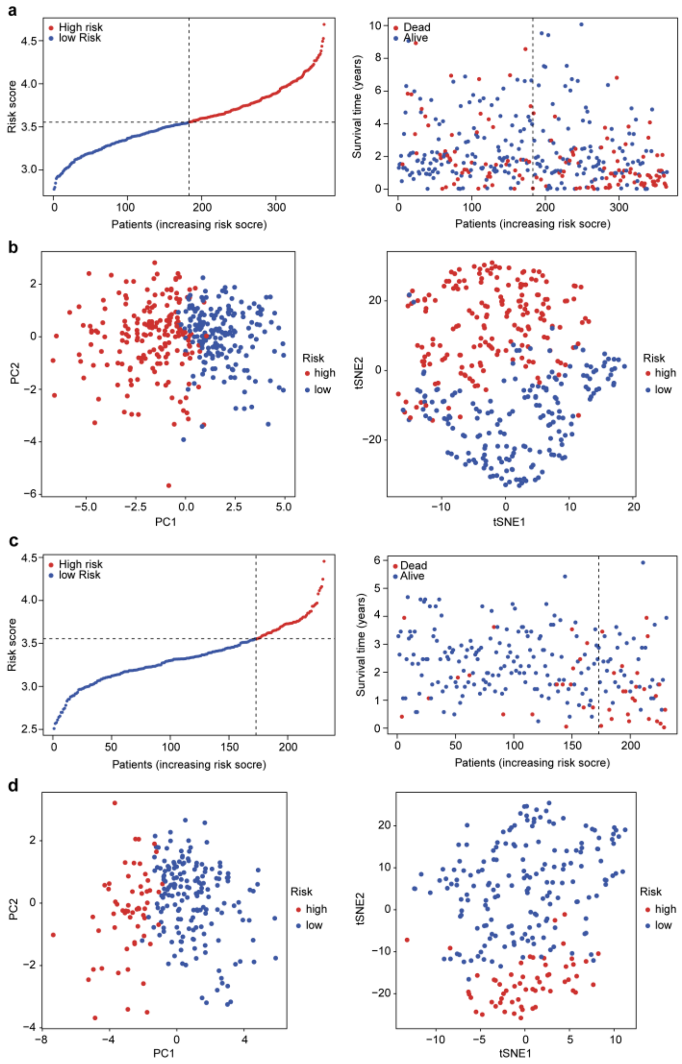

Supplement: Supplementary file 4 [file medi-101-e31814-s004.pdf]
