# Supplementary material for: Identification and validation of immune and prognosis-related genes in hepatocellular carcinoma: A review
Source: Medicine (Baltimore). 2022 Nov 18;101(46):e31814. doi: 10.1097/MD.0000000000031814 (PMC9678506; doi:10.1097/MD.0000000000031814)

Figure S3. Correlations between the risk score and clinical characteristics of patients with hepatocellular carcinoma (HCC)

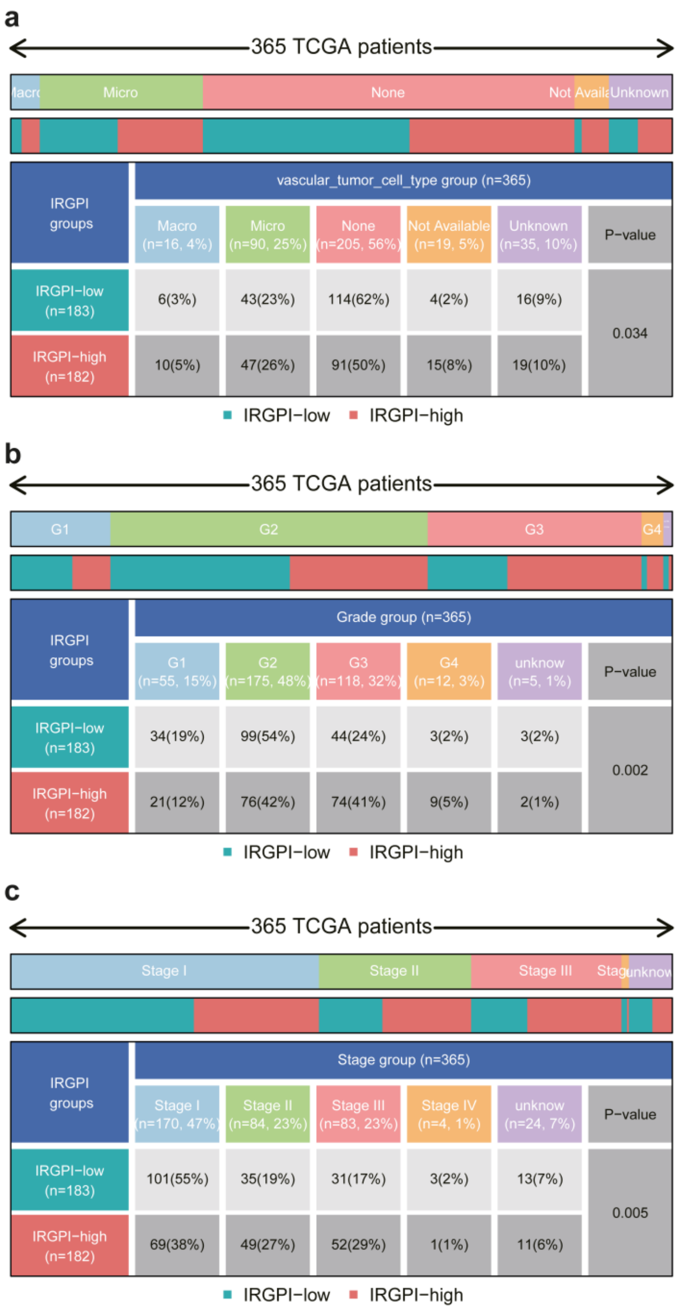

Supplement: Supplementary file 5 [file medi-101-e31814-s005.pdf]
