# Supplementary figures and images for: Identification and validation of immune and prognosis-related genes in hepatocellular carcinoma: A review
Source: Medicine (Baltimore). 2022 Nov 18;101(46):e31814. doi: 10.1097/MD.0000000000031814 (PMC9678506; doi:10.1097/MD.0000000000031814)

**Figure S4.** Correlations between the risk score and overall survival (OS)

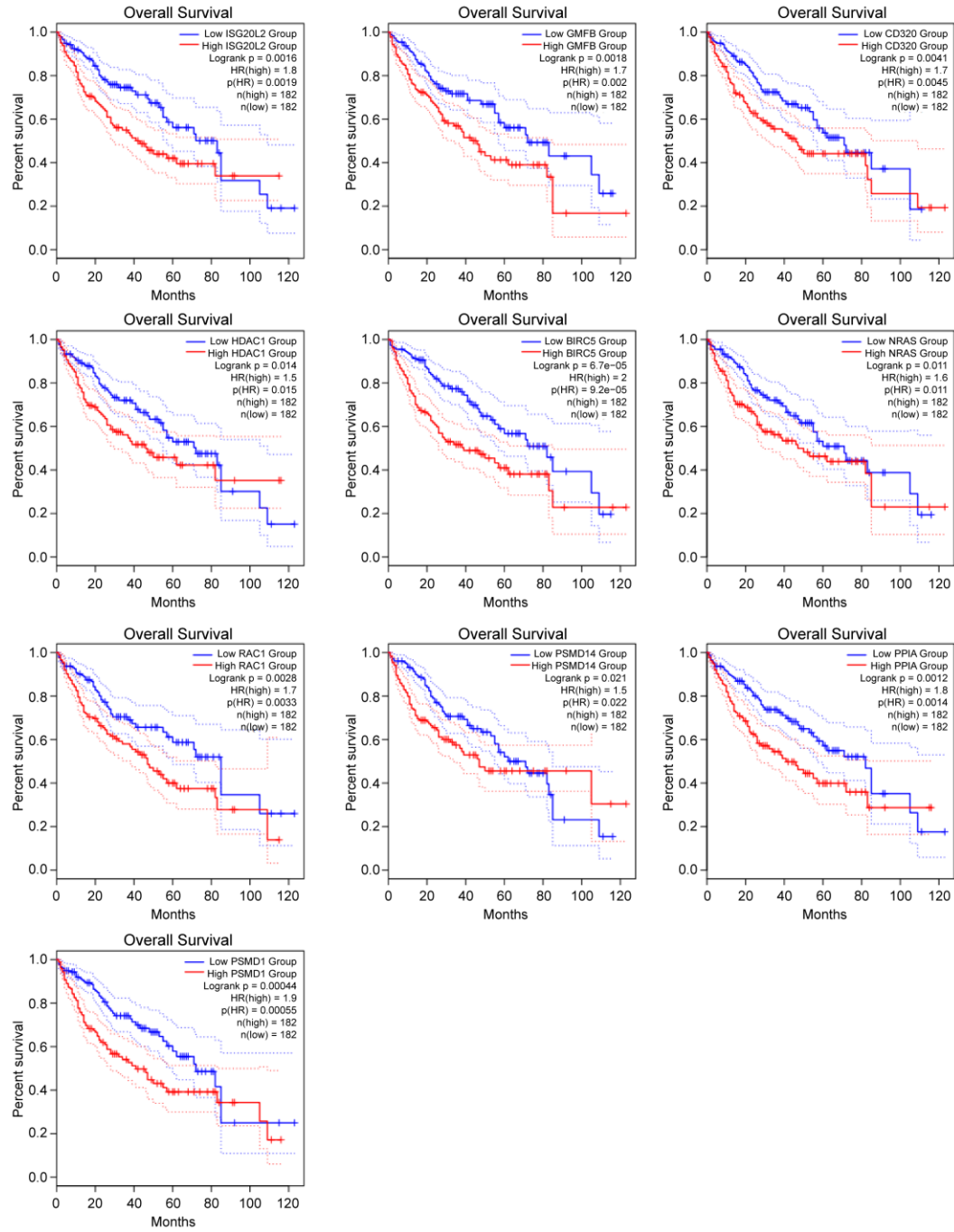

Supplement: Supplementary file 6 [file medi-101-e31814-s006.pdf]

**Figure S5.** Validation of the 10 immune-related hub genes (IRHGs) using external data

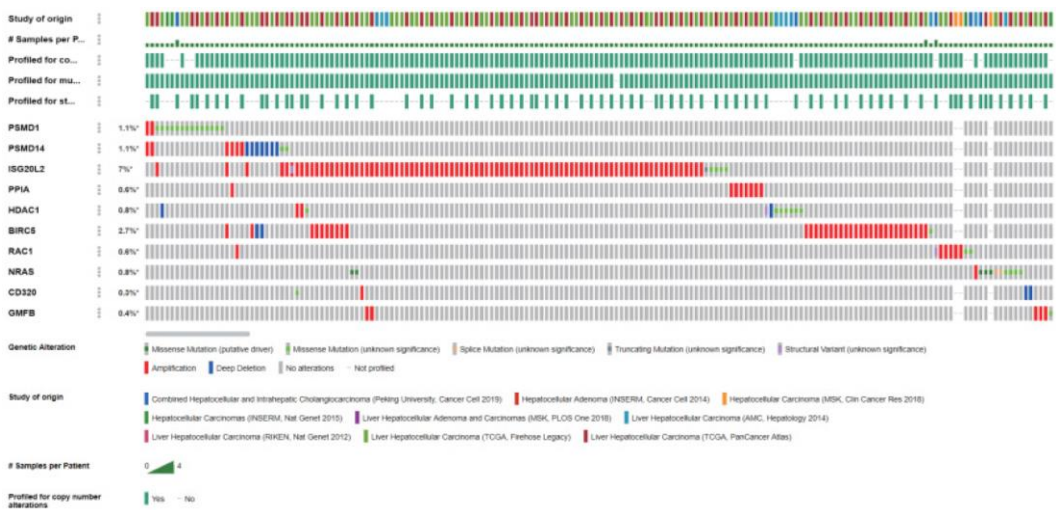

Supplement: Supplementary file 7 [file medi-101-e31814-s007.pdf]
